# Optimizing Aesthetic Facial Surgery Outcomes Following Minimally Invasive Treatments: Guidelines for Perioperative Management

**DOI:** 10.1093/asjof/ojaf087

**Published:** 2025-07-04

**Authors:** Sachin M Shridharani, Melanie D Palm, Theresa Jarmuz, Michael Somenek, Laxmeesh M Nayak, David Saadat, Yula Indeyeva, Jonathan Sykes

## Abstract

**Background:**

The rise in minimally invasive treatments (MITs) in aesthetic medicine has introduced new complexities for subsequent facial surgeries such as facelifts. Despite their popularity, there are limited data on how these treatments impact surgical outcomes.

**Objectives:**

The authors of this paper explore the impact of MIT modalities on subsequent facial surgeries and provide guidelines for perioperative planning and management to optimize outcomes for patients with a history of MITs.

**Methods:**

An expert panel of 7 plastic surgeons and 1 dermatologist conducted a comprehensive review of existing literature, combined with author surveys and case-based discussions, to develop perioperative recommendations for patients with previous MITs. Consensus was reached for each recommendation with a ≥75% agreement threshold.

**Results:**

The authors of this paper present recommendations for perioperative planning, surgical techniques, and postoperative care for patients with previous MITs. Complication risks were found to vary by MIT modality: biostimulatory injectables, temporary fillers, and superficial energy–based devices (EBDs) generally present lower risks, whereas permanent fillers, deeply delivered EBDs, and recently placed temporary fillers or threads were associated with increased risks. The recommendations highlight strategies to support both aesthetic and functional surgical outcomes.

**Conclusions:**

Patients with previous MITs can be candidates for facelift surgery if perioperative strategies are followed to mitigate risks associated with plane distortion and vascular compromise. These guidelines provide a framework to support aesthetic providers in enhancing surgical outcomes and patient satisfaction. Given the limited literature on MIT-related surgical implications, the authors emphasize individualized approaches to mitigate risks associated with previous MITs until further research is available.

**Level of Evidence: 5 (Therapeutic):**

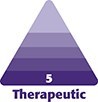

The demand for aesthetic procedures has seen a remarkable and steady increase worldwide. According to data from The Aesthetic Society (formerly the American Society for Aesthetic Plastic Surgery), in the United States alone, over 9 million cosmetic procedures were performed in 2022, encompassing both surgical and minimally invasive treatments (MITs).^1^ MITs have continued to rise in popularity, reflecting a broader trend in aesthetic medicine toward procedures that offer subtle yet significant enhancements with minimal downtime.^2^ In 2022, these treatments outpaced surgical procedures, constituting ∼80% of all cosmetic procedures—a 23% increase from 2021.^1^ MITs include a variety of injectable agents, threads, and energy-based devices (EBDs).

Injectables can be further categorized as neuromodulator injections (botulinum toxin type A [BoNT-A]), temporary biostimulatory injections (poly-L-lactic acid [PLLA], hyperdilute calcium hydroxyapatite [CaHA]), temporary dermal fillers (hyaluronic acid [HA]), unreconstituted CaHA, permanent dermal fillers (polymethacrylate [PMMA], silicone), and adipocytolytic (deoxycholic acid) injections. BoNT-A was the top minimally invasive procedure of 2023, underscoring its role as a well-established MIT.^2^ However, given its relatively short duration of effect, it is unlikely to significantly impact future facial surgeries negatively and will not be addressed in this paper.^3^ HA fillers, closely following BoNT-A in popularity, are widely used alongside a growing demand for non-HA injectables, such as PLLA and CaHA, which stimulate the body's natural collagen and elastin production for long-term skin rejuvenation.^2^

Threads used in aesthetic procedures can be categorized as absorbable (eg, polydioxanone [PDO], PLLA, and polycaprolactone) or nonabsorbable (eg, polypropylene), with absorbable threads increasingly favored because of their gradual tissue integration over time and decreased risk of complications.^4^

EBDs encompass various purposes and devices, categorized herein by depth of effect. EBDs that deliver energy to Layer 1 (dermis and epidermis) and/or Layer 2 (subcutaneous fat) are considered superficial EBDs, whereas deeply delivered energy is defined as energy delivered to Layer 3 (superficial musculoaponeurotic system [SMAS] and muscle). Superficial EBDs encompass microneedling, micro-coring, lasers, ultrasound, helium plasma dermal resurfacing, and cryolipolysis; EBDs such as subdermal radiofrequency (RF) and helium plasma are considered deeply delivered energy. High-intensity focused ultrasound (HIFU) can be superficial, deep, or both. Frequently utilized EBDs for facial and neck rejuvenation include skin resurfacing lasers, HIFU for reducing wrinkles and enhancing skin firmness, cryolipolysis for fat reduction, and RF-based devices for skin tightening and collagen contraction.^2,5^ RF-assisted liposuction (RFAL) and RF/helium plasma devices induce soft tissue contraction and collagen stimulation through the delivery of heat to the subcutaneous and fibroseptal network.^6^ The controlled thermal injury induces coagulation of subcutaneous fat, contraction of collagen fibers in the subcutaneous tissues, and formation of scar tissue within the fibroseptal network, thereby reducing skin laxity.^6,7^ The use of noninvasive skin tightening methods has increased by 9% since 2022.^2^

Despite the data underscoring the growing popularity and widespread acceptance of MITs as part of the broader landscape of aesthetic care, there remains a paucity of data regarding their long-term implications, particularly in the context of subsequent surgical interventions such as facelifts. The authors affirm that biostimulatory treatments rarely result in complications that cause challenges in performing subsequent aesthetic facial surgery; with appropriate planning and risk management, patients who have received previous MITs are still candidates for future aesthetic facial surgery. The aim of the authors of this paper is thus to offer a comprehensive understanding of the impact of MITs on subsequent facelift surgeries and to provide evidence-based recommendations to guide patients and practitioners in making informed decisions, enhance perioperative planning, and optimize patient outcomes. Although based on available data, indirect evidence, and the expert opinion and experience of the author panel, it is imperative for aesthetic treatment providers to carefully assess the risks and benefits of the techniques recommended herein, taking into account each patient's medical and treatment history, preferences, needs, and overall suitability.

## METHODS

An expert multidisciplinary panel is composed of 7 plastic surgeons who cumulatively have over 75 years of experience, who perform both face lifts and use MITs in their patients, and 1 dermatologist, who has over 10 years of dermatology experience including treating patients daily with MITs. Additionally, these panelists have published hundreds of peer-reviewed articles, served as Key Opinion Leaders and regularly present at national and international conferences. These panelists assembled to develop recommendations on the impact of MITs on subsequent aesthetic facial surgery. Experts were selected to be part of the panel based on their medical expertise, experience, and familiarity of MITs. The authors identified 3 guideline questions:

1. What should be considered when using MITs in patients who may pursue aesthetic facial surgery in the future?
2. What are the implications of previous MITs for facial surgery?
3. What perioperative strategies could help mitigate the potential challenges caused by MITs?

**Figure 1.**
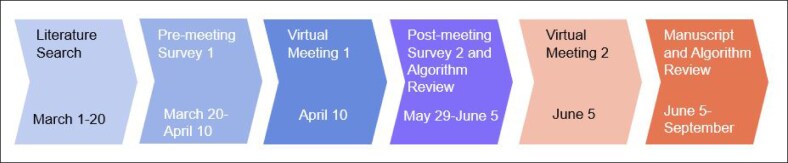
Overview of the consensus process.

A set of recommendations and a treatment algorithm with guidance on perioperative strategies to minimize or mitigate risks were formulated through literature reviews conducted by the meeting facilitators before the virtual meetings, author premeeting and postmeeting surveys, and case-based discussions across 2 virtual meetings (Figure 1). The expert panel then used a 5-point Likert scale to vote on each recommendation after the first virtual meeting via a postmeeting survey, with acceptance defined as ≥75% agreement. Faculty responses were summarized using mean values on a 5-point Likert scale, in which 1 indicated strong disagreement and 5 indicated strong agreement.

### Recommendations

Aesthetic providers and patients should be aware that the risk of complications from MITs varies by modality (Table 1). According to the panel, HA fillers are least likely to cause issues. Compared with HA fillers, biostimulatory injectables (eg, PLLA, hyperdilute CaHA), temporary fillers (eg, unreconstituted CaHA), and superficial EBDs (eg, ultrasound) pose a somewhat higher risk of dissection difficulty. In contrast, permanent injectables (eg, PMMA) and recently placed absorbable threads (within 6–9 months before facial surgery) pose higher risks again. The panel also agreed that deeply delivered EBDs (eg, RF/helium plasma) affecting the subdermal and fibroseptal network present the greatest risk to future facial surgery outcomes. Although most experienced plastic surgeons are comfortable performing surgeries through careful consideration and risk assessment, whether it is because of previous surgical procedures or previous MITs, some challenges may arise, such as fusion of the glide planes after treatment with deep energy-based treatments.

**Table 1.** Recommendations for Question 1: What Should be Considered When Using Minimally Invasive Treatments in Patients Who May Pursue Aesthetic Facial Surgery in the Future?

Recommendations for patients receiving minimally invasive treatments who are likely to pursue subsequent facial surgery	Level of agreement
Treatment plans should be developed in consideration of the treatment location and modality, in order to minimize potential complications and optimize outcomes in patients pursuing MITs who may be future surgical candidates	4.9/5
Aesthetic treatment providers and surgeons must inform patients of the potential impact of MITs on future facial surgeries	4.7/5
Aesthetic treatment providers and patients should understand that the likelihood that an MIT might cause complications for facial surgery varies by treatment type: Temporary biostimulator injections (eg, PLLA, hyperdilute CaHA), temporary fillers (eg, HA, unreconstituted CaHA), and superficial-acting EBDs (eg, ultrasound) are the least likely to cause complications when used as indicated in defined facial locations Permanent injectables (eg, PMMA) and threads and recently placed (ie, 6-9 months before surgery) temporary injectables and absorbable threads are more likely to cause complications Deeply delivered EBDs (eg, RF/helium plasma) that affect the subdermal and/or fibroseptal network are the most likely to cause complications	4.3/5
Injectable treatments should be administered sufficiently in advance of facial surgery to allow for optimal product maturation and resolution of inflammatory processes by the time of the operation	4.7/5
Injectables must be administered in the appropriate volume and plane as described in the product monograph for the specific product: Injections should avoid crossing distinct tissue planes Injections should be administered to the supraperiosteal and/or subcutaneous planes, as indicated for the defined facial locations, to minimize the likelihood of complications Injection into the SMAS or intramuscular planes may interfere with facial aesthetic surgery Products must be evenly distributed, avoiding the injection of boluses and excess volume (ie, overfilling)	4.9/5

CaHA, calcium hydroxylapatite; EBDs, energy-based devices; HA, hyaluronic acid; MIT, minimally invasive treatment; PLLA; poly-L-lactic acid; PMMA, polymethyl-methacrylate; RF, radiofrequency; SMAS, superficial musculoaponeurotic system.

### Complications With Injectables

The overall incidence of long-term adverse events (AEs) following injectable treatment is low, with most cases involving serious AEs linked to administration errors.^8,9^ Across products, overfilling and administration into the incorrect area or across planes can, in rare cases, cause chronic inflammation, fibrosis or hypertrophic scarring, nodule/granuloma formation, edema, induration, delayed healing, and compression of surrounding vessels, leading to localized skin necrosis.^10-13^ The physicochemical properties of dermal fillers, such as hydrophilicity, viscosity, crosslinking, and concentration, can also contribute to poor filler distribution and create undue pressure on surrounding tissues.^13,14^ This pressure can lead to stretching, thinning, and weakened structural integrity of the skin and underlying tissues when overinjection occurs.^13,14^

**Figure 2.**
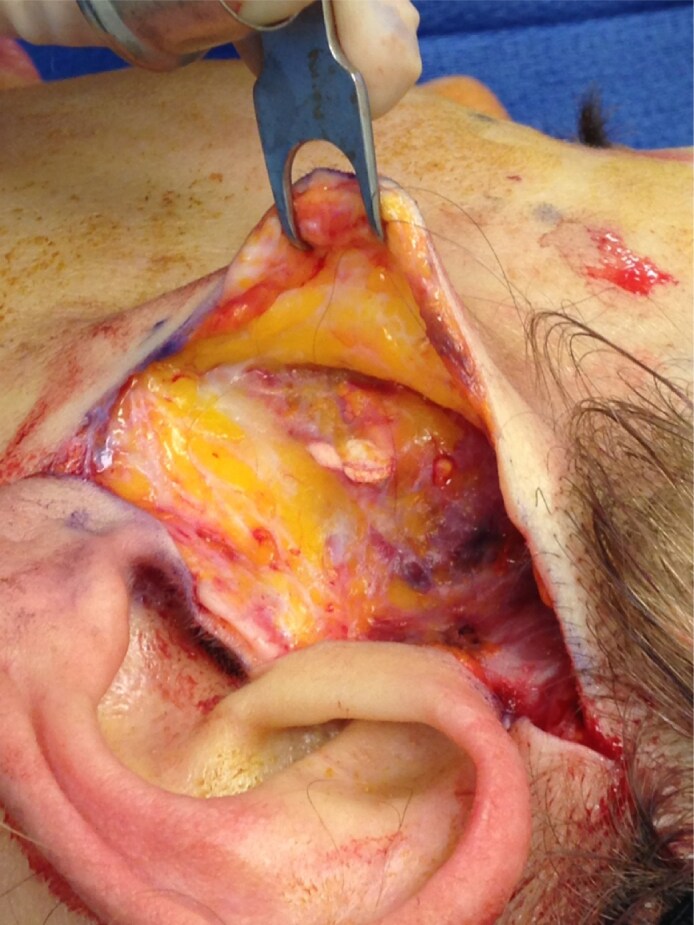
Image of a calcified nodule from a 55-year-old female patient 24 months status post calcium hydroxyapatite injection.

When used correctly, HA fillers enhance facial skin quality through dermal hydration, and, like other temporary fillers (eg, unreconstituted CaHA), pose minimal risk to future facial surgeries.^15^ Reactions with temporary dermal fillers are usually mild and transient, such as erythema, edema, bruising, and pain.^16,17^ More severe outcomes such as nodule formation and vascular occlusion are less common (Figure 2).^16,17^

Biostimulatory injectables, such as PLLA, hyperdilute CaHA, and intradermal microinjections of cross-linked HA, have a low likelihood of complicating future facial surgeries given their low potential for inducing Type III (fibrotic) collagenesis.^18-22^ Their stimulation of Type I collagen, combined with the suppression of elastin degradation, aligns with natural remodeling processes and minimizes the risk of rigid, inelastic fibrotic tissue, and associated surgical challenges.^18-23^ Granulomas and nodules are rare with biostimulators, with nodules reported in <1% of patients in a retrospective case study of patients receiving PLLA injections.^24^ Nevertheless, they are among the more commonly reported complications of biostimulatory particle-containing injectables, attributable to the use of higher filler volumes and concentrations, larger bolus injections, frequently repeated treatments, and inappropriate injection techniques.^16,25-30^

In contrast, permanent fillers such as PMMA and silicone pose higher risks for future surgery. Complications from these modalities are also rare, but they have been associated with granulomatous reactions and persistent nodules.^31-33^ Other rare complications from permanent fillers can be severe and long-lasting, including capsular contracture, filler migration or extrusion, eyelid malposition, tissue hardening, and chronic inflammation.^31-33^

When used to reduce submental fat, the most commonly reported AEs with adipocytolytic injections include transient mild-to-moderate swelling, hematoma, bruising, and induration.^34^ However, the adipocytolytic action of these injections can promote complications such as skin necrosis, nerve lesions, vascular events, fibrosis, and nodules, all of which may affect the success and safety of future facial surgeries.^34^

### Complications With Threads

The most frequently observed complications of threads are transient swelling and skin dimpling, which have been reported in ∼33% and up to 35% of all patients, respectively.^35,36^ Dimpling typically resolves spontaneously, although long-standing dimpling is a rare but reported occurence.^35^ Patients older than 50 years are particularly susceptible, exhibiting a significantly higher risk of dimpling (16% vs 5.6%) and infections (5.9% vs 0.7%) compared with their younger counterparts.^35^ Edematous tissue can impair stable thread anchoring, which can lead to rare, more severe complications such as thread visibility, palpability, extrusion, or displacement.^35^

Thread extrusion, visible threads, skin dimpling, and irregular contours have been reported as the most common AEs of facial thread lifting for which patients seek removal.^37^ Absorbable threads are associated with a significantly lower risk of extrusion than permanent threads (1.6% vs 7.6%).^35^

Rare chronic complications can involve persistent inflammation, subcutaneous induration, granuloma, abscess, scarring, contour irregularities, loss of skin integrity, dynamic skin and soft tissue tethering, and asymmetry.^35,36^ These complications are often attributable to improper technique and can complicate future facial surgeries.^36,37^

### Complications With Superficial Energy–Based Devices

AEs associated with superficial EBDs, such as microneedling, micro-coring, and superficial laser treatments, are typically confined to the dermis and, as such, are generally regarded by the authors as less likely than deeply delivered EBDs to impact the outcomes or complexity of future facial surgery. Nevertheless, improper device settings or technical errors can extend treatment effects to deeper tissues, increasing the risk of complications that may affect surgical outcomes.

Superficial laser aesthetic treatments are associated with a relatively low complication rate, because their effects are typically confined to the dermis.^38^ However, complications can occur, with the most common being temporary erythema and edema.^39^ Inappropriate operation and poor technique can result in more serious complications, such as persistent erythema because of extensive residual thermal necrosis, or delayed wound healing, which increases susceptibility to infections.^38,39^ In rare cases, hypertrophic scarring may arise several months after laser application from infection or excessive thermal injury.^38,39^ Additionally, eruptive keratoacanthomas have been reported in 6 cases following laser resurfacing.^38,40^

Microneedling and micro-coring are generally well tolerated, but improper technique, such as incorrect needle depth or excessive overlap, can result in crusting, petechiae, dermal scarring, burning, postinflammatory hyperpigmentation (PIH), and granuloma formation lasting for several days to weeks.^41^ A recent systematic review of the literature on granulomatous reactions from microneedling found that most cases are attributed to factors such as the application of topical agents during the procedure, particularly Vitamin C serums, and the use of motorized microneedling pens.^42^ Additionally, the introduction of immunogenic particles into the dermis, such as silica-containing products applied either pre- or postprocedurally during microneedling, can result in granuloma formation by triggering a granulomatous hypersensitivity reaction.^43^

Evidence suggests that the micro-cores removed during micro-coring are typically too small to cause scarring in most individuals.^44^ However, patients with darker skin tones or a history of keloid formation may be at risk following microneedling or micro-coring.^45^ Fitzpatrick skin Types IV to VI are also more prone to PIH and scarring because of increased melanin production.^45^ Although PIH does not directly cause scarring, inflammation can lead to complications such as dyspigmentation, keloids, and hypertrophic or atrophic scars, especially with severe injury or improper healing.^45^

RF-assisted micro-coring and microneedling have also been shown to increase epidermal thickness and reduce skin surface area and/or volume.^41^ Additionally, RF microneedling induces thermal coagulation injury at the needle penetration depth; depending on the depth of the needle, this could impact deeper tissue planes.^41^

### Complications With Deeply Delivered Energy-Based Devices

Complications influencing subsequent facial surgery are more prevalent in patients treated with deeply delivered EBDs, such cryolipolysis, RFAL, RF/helium plasma, and HIFU. The most frequently reported AEs associated with cryolipolysis include erythema, numbness/paresthesia, bruising, and edema at the treatment site, all of which are generally mild and resolve without intervention.^46^  ^47^ Rare but serious AEs include fibrosis, visible contour irregularities, skin hyperpigmentation, and paradoxical adipose hyperplasia (PAH).^46,47^ Although the exact pathophysiology of PAH remains unclear, it is believed that a reactive fibrotic process in response to damaged adipocytes leads to fat hyperplasia, thickened fibrous septae, painful swelling, and firm mass formation—factors that may complicate surgical contouring.^46,48,49^ Because condensation via RF and/or helium plasma is not tissue specific, in addition to the target fat and connective tissue, coagulation of vasculature, nerves, and lymphatics may also occur.^50^ Although uncommon, this can result in changes to the SMAS layer, vertical scarring across tissue planes, symphysis between the skin and the muscle, densely adherent platysma fascia, thermal-induced shorting, fibrotic subcutaneous bands, cheek fat atrophy, nodules, and induration of subcutaneous tissue.^7,36,50^ EBD-induced thermal collagen contraction and remodeling, particularly within the fibroseptal network, can also lead to a retraction of soft tissue flaps.^51^ Volumetric analysis has shown soft tissue contraction of up to 47% following RFAL, potentially contributing to thinner flaps in these patients.^51^ Seromas, likely resulting from injury to the subcutaneous and subdermal lymphatic system, along with prolonged skin thickening and brawny edema from thermal treatments, may take several months to fully resolve.^50^ Additionally, in a review of 207 rhytidectomy patients from 2012 to 2017 who had previously used MITs, 7% had cheek fat atrophy and 13% had significant scar tissue formation because of EBDs.^36^

In their experience, the authors find that complications from MITs that distort tissue planes and compromise vascularity are the most significant factors impacting the success of subsequent aesthetic facial surgery (Table 2).

**Table 2.** Recommendations for Question 2: What are the Implications of Previous Minimally Invasive Treatments on Facial Surgery?

Recommendations related to possible facial surgery challenges post minimally invasive treatments	Level of agreement
Although complications are rare post MITs, they occur often enough that surgeons should be aware of and prepared for potential complications that might be encountered during facial surgery, in particular: Distortion of tissues, leading to challenges in identifying vital structures, such as native neurovasculature, and difficulty separating planes to develop healthy flaps Compromised vascularity, leading to challenges with flap viability and longevity of effect	4.9/5
Surgeons should be aware that certain highly treated areas are more susceptible to surgical complications based on the type of previous treatments received: It may become difficult to develop tissue flaps following injections above the jawline It may become difficult to develop tissue flaps following lipolysis treatments or deeply delivered EBDs below the jawline	4.6/5

EBDs, energy-based devices; MIT, minimally invasive treatment.

### Implications of Fibrosis

The impact of fibrosis on facial surgery, particularly following MITs, has become a significant topic of discussion. However, it is crucial to clarify that the primary concern for experienced facial surgeons lies not merely in the presence of scarring or fibrosis but in the alterations to tissue planes that may ensue.

For example, although facelifts do result in scar tissue, this tissue develops along, not across, surgical planes and is typically limited to the subcutaneous plane, which poses fewer challenges for revision surgeries. The deeper planes remain largely unscathed, continuing to function as glide planes for surgical manipulation.

The real challenge with fibrosis arises when the characteristic overproduction and accumulation of extracellular matrix components impact tissue function and cause architectural distortion.^52^ It is particularly challenging when tissue planes are distorted; for example, when deeply delivered EBD treatments cause certain areas, such as the preauricular skin, to adhere tightly or develop vertical scarring across planes. This tethering of tissue layers can lead to rigidity, impeding the natural movement of tissues and exacerbating plane distortions.^50,53-59^ Furthermore, chronic fibrotic and inflammatory responses may lead to tissue thickening, hardening, vascular changes, and edema, with the latter potentially causing atrophy, necrosis, and contour depressions, all of which contribute to further plane distortion and negatively impact postoperative aesthetic outcomes.^60,61^

### Implications of Plane Distortion

Fibrosis, tissue contraction and remodeling, adhesions, nodules, granulomas, and inflammation can all contribute to plane distortion. During surgical procedures, these distortions can obstruct the surgeon's ability to clearly identify and separate tissue planes, making it challenging to develop flaps and accurately dissect structures, increasing the risk of complications and suboptimal outcomes. Abnormal tissue architecture, stickiness or anchoring of tissue layers to each other, and fibrotic changes can result in increased stiffness, complicating flap elevation and mobility, as well as suturing, particularly in the SMAS layer. Distortions in surgical planes further increase surgical complexity, oftentimes necessitating additional intraoperative strategies and occasionally precluding safe completion of the full original surgical plan. Deeper nodules or granulomas may remain untreated or concealed until facelift surgery, especially in patients with ptotic skin or dense subcutaneous tissue.^62^ During surgery, these irregularities can compound plane distortion challenges, complicate dissection, and create visible contour asymmetries.^62^ If not adequately corrected, these issues can result in aesthetically displeasing outcomes, ultimately reducing patient satisfaction and the overall efficacy of the surgical treatment. Finally, the longevity of the procedure may be reduced by the need to use alternative techniques required to manage fused planes. Although these observations are based on the authors’ clinical experience, there is limited literature addressing the implications of plane distortion from MITs on surgical outcomes, underscoring the need for further research to better understand and optimize management strategies in this context.

### Implications of Vascular Compromise

Although vascular occlusion from fillers is an acute condition occurring in <0.02% of injections, it can have a protracted impact on surrounding tissues; prolonged ischemia with insufficient collateral flow increasingly compromises tissue integrity, posing a risk to deeper structures.^63^ Reperfusion, although essential to restore oxygen, can induce metabolic distress and trigger an excessive immune response, leading to reperfusion injury.^64^ This process generates free radicals, setting off a cascade of cellular events that promote inflammation and cause endothelial dysfunction, DNA damage, and ultimately necrosis or apoptosis.^64,65^ These effects disrupt cellular function, degrade extracellular matrix proteins, and impair dermal fibroblast and keratinocyte function, compromising tissue integrity and hindering healing in the affected area.^64,65^

Additionally, tissue distortion from chronic inflammation, nodules, granulomas, or tissue adherence can impinge on blood vessels, potentially resulting in long-term lymphatic and vascular compromise.^30,36,66^ Prolonged extravascular compression from these complications or injected materials can reduce perfusion, resulting in chronic ischemia and weakening tissue integrity.^67^ Friable tissues may not respond well to surgical manipulation because of their inability to withstand tension and maintain closure, increasing the risks of graft failure, poor tissue integration, wound dehiscence, infection, and scarring. This is especially critical in cosmetic procedures, in which aesthetic outcomes are paramount.

Based on literature and expert opinion, the authors confirm that patients with a history of MIT treatments can remain candidates for future aesthetic facial surgery. They propose perioperative strategies (Table 3) and an algorithm (Supplemental Figure 1) to mitigate complications and optimize patient outcomes.

**Table 3.** Recommendations for Question 3: What Perioperative Strategies Could Help Mitigate the Potential Challenges Caused by Minimally Invasive Aesthetic Treatments?

Recommendations for perioperative facial surgery post minimally invasive treatments	Level of agreement
The following “preoperative” strategies are “recommended”: Obtain a detailed treatment and medical history focused on the use of previous minimally invasive treatments Conduct a thorough physical examination to identify any possible impacts because of previous treatments • Inspect skin for the presence of nodules, lymphatic and/or vascular compromise • Confirm normal mobility, animation, structure, and musculature Discuss possible intraoperative complications with the patient and secure preemptive consent for alternative approaches/management of complications Ensure sufficient time since last minimally invasive treatment Dissolve fillers in affected areas at least 2 weeks before surgical intervention if symptomatic/pathologic, present in surgical planes, or cause distortions	4.9/5
The following “preoperative” strategies should be “considered”: Plan for an extended operation time to accommodate potential challenges Administer hyperbaric oxygen for high-risk patients (ie, those with compromised vascularity, significant scarring, and/or previous facial surgery complications)	4.7/5
The following “intraoperative” strategies are “recommended”: Remain vigilant for physiological and anatomical findings Be prepared to pivot surgical strategies	5.0/5
The following “intraoperative” strategies should be “considered”: For patients who have been previously treated with EBDs, inject nanofat subcutaneously For patients who present with dermal scarring, develop thicker adipocutaneous flaps Inject growth factor beneath flaps at closure	4.0/5
Although standard postoperative care applies, the following “postoperative” strategies should also be “considered”: Advise patients to remain nearby for more frequent monitoring and symptom management following the surgical procedure Systemic steroids, or other immunomodulators, to control the inflammatory response Hyperbaric oxygen therapy for patients presenting with vascular compromise and excessive fibrosis	4.6/5

EBD, energy-based device.

### Preoperative Strategies

#### Medical History

Obtaining a thorough medical history is essential for developing an effective perioperative plan, particularly in facial surgery. Understanding past aesthetic treatments, especially procedures involving deeply delivered energy, is crucial, because these can significantly influence surgical outcomes. Additionally, it is important to determine whether the patient has a history of vascular occlusion or ischemic events, which may be more likely depending on the modality of previous treatments. Patients may not recall the true nature of previous complications and often fail to mention them during surgical consultations.^62^ Thus, it is essential to inquire specifically about any past treatments and skin discoloration, because these may indicate previous vascular issues.^62^ Knowing the location of such injuries is critical, because they can affect the vascularity of skin flaps and increase the risk of postoperative wound healing complications.^62^

Surgeons should also be mindful of risk factors such as previous complications with fillers, multiple treatments, poor hygiene, immunocompromised status, chronic skin conditions, smoking, second-hand smoke, vaping and use of nicotine products, and uncontrolled diabetes.^16^ Additionally, recent facial trauma, extensive dental work, or vaccinations could trigger a delayed hypersensitivity reaction and should be carefully evaluated.^16^ In some cases, postponing surgery may be necessary if the patient is unwell (eg, fever), to ensure optimal outcomes and minimize risks.

#### Patient Education and Consent

Thorough patient education on the potential intra- and postoperative risks associated with surgical intervention following MITs is crucial. Effective patient education not only enhances informed decision making, but has also been shown to reduce postoperative complications, decrease pain medication use, and improve adherence to postsurgical care instructions.^68^ Empowered patients often experience better quality of life and treatment satisfaction, demonstrate improved self-management, and may contribute to lower healthcare costs by reducing the need for additional follow-up procedures and interventions.^68^

Additionally, because changes to the surgical plan may be necessary to adapt to unexpected findings and optimize outcomes, obtaining preemptive consent for intraoperative alterations to surgical techniques, including the injection of nanofat and/or growth factors, is essential.

#### Nanofat Injections

In the rare cases in which the use of MITs has resulted in extensive fibrosis and irregular contours, the authors suggest considering the use of autologous nanofat injections. Adipose-derived stem cells in nanofat secrete factors that promote myofibroblast apoptosis (soften fibrotic tissues), reduce inflammation, and enhance vascularization and skin physiology.^69^ Nanofat has also been shown to support subtle contour improvements, aid angiogenesis, and potentially contribute to graft integration and improved skin texture.^69-71^ Although studies on nanofat are limited in sample size and rely on subjective outcomes, this approach, when used intraoperatively, may increase the pliability of dense, fibrotic tissue and may be particularly beneficial for thin flaps with compromised blood supply and limited volume, enhancing both postoperative healing and patient satisfaction.^69^

Notably, however, high rates of fat resorption have been observed when treating fat atrophies attributable to EBDs, potentially reducing the effectiveness of nanofat and necessitating additional treatments, although this can be technique- and patient-dependent.^36,72^

### Preoperative Hyperbaric Oxygen Treatment

In high-risk patients with compromised vascularity, significant fibrosis, or a history of facial surgery complications, preoperative hyperbaric oxygen treatment (HBOT) should be considered at the discretion of the operating surgeon. HBOT reduces inflammation, stimulates angiogenesis, and enhances the activity of key immune cells, improving tissue oxygenation.^73,74^ It has proven effective in treating various disorders characterized by compromised tissue oxygenation and perfusion, such as microvascular diabetic complications, radionecreosis, filler-induced vascular occlusion, and other areas of aesthetics.^73,75-80^ Preoperative HBOT has shown benefits in reducing ischemia-reperfusion injury in animal models and has been associated with decreased intraoperative blood loss, shorter postoperative stays, fewer complications, and reduced costs in clinical studies.^73,81,82^ Additionally, the authors note that preoperative HBOT can facilitate smoother tissue plane separation during facial surgery by reducing fibrosis and improving tissue elasticity, thereby easing surgical manipulation.^76^

### Operative Timing

Proper timing of facial surgery in patients who have undergone minimally invasive aesthetic treatments is critical to minimize complications and ensure optimal surgical outcomes. Different treatments have varying impacts on tissue structure and healing processes, necessitating specific waiting periods before performing facial surgery.

The authors generally advise against performing facial surgery within 2 weeks of treatments that may cause inflammation. Residual inflammation or edema can render the tissues stiff and difficult to separate, potentially increasing the risk of excessive intra- and/or postoperative bleeding or fluid accumulation.^83^

#### Dermal Fillers

Dermal fillers and biostimulatory injections should be administered sufficiently in advance of facial surgery to allow for optimal maturation of the product and neocollagenesis by the time of the operation. Although HA dermal fillers have limited longevity and are biodegradable, they often persist in tissues longer than specified in product monographs.^79-81^ Given that hyaluronidase can continue to degrade HA fillers gradually over up to 2 weeks, it is recommended to administer hyaluronidase at least 2 weeks before facial surgery for patients with a history of HA fillers, particularly in the infraorbital hollow(s) and areas of topographic concern.^15,84,85^

#### Absorbable Threads

The authors recommend a waiting period of 6 to 9 months after initial implantation of absorbable (PDO, PLLA) threads before performing facial surgery. Temporary threads dissolve ∼6 months after implantation; however, thread-induced collagen synthesis may continue for another 2 to 3 months.^86^ Allowing adequate time for the threads to dissolve and collagen to mature reduces the likelihood of tissue distortion and/or neurovascular obstruction by residual thread material or ongoing collagen production. This is especially important if threads have been placed in or migrated to the sub-SMAS near facial nerves, to minimize the risk of accidental damage to these structures. Prompt thread removal may be warranted with any subsequent facial procedure in cases in which complications or patient discomfort occurs, as long as the threads are not wrapped around or lodged directly with any neurovascular bundle.

#### Adipocytolytic Injections

Studies show that fibroblast migration completes by the 28th day post treatment with deoxycholic acid injections.^34,87^ However, continued fibroblastic responses have been observed for several months after the last injection. Therefore, it is recommended to schedule facial surgery at least 1 month after the last deoxycholic acid treatment, ideally waiting 6 months.

#### Cryolipolysis

Although the postcryolipolysis inflammatory process typically peaks between 2 and 4 weeks after treatment, lipid metabolism and excretion can take 3 to 4 months to complete.^88,89^ Additionally, PAH generally manifests 2 to 3 months post treatment, and its management is advisable only after the affected area has softened, typically within 6 to 9 months of the initial procedure.^90,91^ Consequently, it is recommended to schedule facial surgery no earlier than 4 months after the final cryolipolysis session, with further delays warranted in cases of PAH.

#### Superficial Lasers

After laser therapy, the healing process follows distinct stages, each with its own timeline. Although surface healing is typically rapid, ongoing improvements can be observed for up to 3 to 6 months as collagen continues to remodel and mature.^92^ Notably, around 3 months after laser resurfacing, lifting effects in photoaged skin are likely attributable not only to new collagen deposition but also to residual edema.^92^ Therefore, it is advisable to wait at least 3 months for full re-epithelialization, edema resolution, and initial collagen maturation before performing facial surgery.

#### Microneedling and Micro-Coring

Data on the long-term and precise duration of effects of micro-coring are currently limited. However, the effects of microneedling (without RF), such as increased skin thickness, tropoelastin, and collagen, occur 3 months following treatment, although the most significant volumetric changes occur at 6 months.^93^ It is thus advisable to wait at least 6 months following microneedling treatment before attempting facial surgery.

#### Radiofrequency-Assisted Therapies

Studies indicate that significant increases in collagen production can be observed over the first 3 months following subdermal coagulation with RF, but the process of collagen maturation and integration into the existing matrix can continue for up to 12 months.^94,95^ Allowing for this extended period of dermal remodeling is recommended before considering any additional surgical procedures.

### Intraoperative Strategies

#### Flap Thickness

Dermal scarring, nodules, and/or fused or distorted planes can increase the risk of vascular compromise and fibrosis, resulting in denser, less elastic tissue that complicates surgical procedures. Depending on the specific planes that are fused or accessible, surgeons might consider switching to a deep plane dissection or performing an extended subcutaneous dissection with plication to optimize outcomes and reduce the risk of complications.

In patients presenting with extensive dermal scarring and/or unresolvable nodules, development of thicker adipocutaneous flaps should be considered. The additional subcutaneous tissue increases blood supply, reducing the risk of postoperative ischemic complications, ensuring better integration and survival of the flap.^96^ Furthermore, thicker flaps provide a cushioning effect, distribute tension more evenly, and minimize the impact of underlying scar tissue, thereby improving the immediate postoperative appearance and long-term outcomes.^96,97^ However, the thicker flap may limit the operating space of the SMAS dissection.

However, when difficulties arise in identifying neurovascular structures, a more conservative operation, such as SMAS plication, may be advisable over a deep plane dissection, because this approach minimizes the risk of damaging critical structures.

Regardless, the choice of technique should ultimately focus on achieving optimal results, rather than on presumed complication rates. This is supported by evidence from a 2019 meta-analysis that found that, although deep plane facelifts had a statistically higher incidence of hematomas compared with less invasive techniques such as SMAS plication, overall complication rates of all rhytidectomy techniques were low and clinically insignificant.^98^

#### Hyaluronidase

Hyaluronidase temporarily disrupts normal extracellular matrix architecture, and thus, flooding with hyaluronidase can facilitate easier dissection through subcutaneous tissue planes, minimize the loss of surface contour, and aid in resolving problematic nodules, granulomas, and HA filler that may be encountered during the procedure.^85,99^

Additionally, pretreatment with hyaluronidase 30 min before fat grafting demonstrated increased vascularity, reduced inflammation, and improved volume retention and survival of the fat graft in an animal model.^100^

#### Growth Factor Injections

Growth factors (eg, platelet-derived growth factor released by platelet-rich fibrin and platelet-rich plasma) have been shown to be a potent tool in cosmetic, plastic, and reconstructive surgery, enhancing wound healing and improving surgical outcomes.^97-99^ By initiating a coagulation cascade, increasing dermal collagen levels, and activating fibroblasts, growth factors support angiogenesis, ameliorate scarring, improve flap and fat graft survival, and accelerate re-epithelialization.^101^ This approach may reduce operating time, postoperative pain, and the need for drains and pressure dressings through lowering rates of hematoma and edema.^101^

### Postoperative Strategies

Surgery carries inherent risks of potential complications; these risks may be particularly pronounced in patients who have previously undergone MITs. Previous reactions to MITs provide critical insights into potential postsurgical outcomes and may predispose these patients to postoperative complications.

In patients previously treated with EBDs, particularly deeply delivered energy, prolonged wound healing, hypertrophic scarring, and postoperative ischemia and necrosis of skin and soft tissue have been observed.^36,102^ Additionally, prolonged postoperative swelling, attributable to lymphatic blockage caused by fillers, has also been observed in patients who have received multiple injectable treatments.^36^

Although general postoperative care standards apply, monitoring of this patient population should be highly individualized and the authors recommend more frequent follow-up visits and closer monitoring, at minimum.

### Postoperative Hyperbaric Oxygen Treatment

Prolonged and persistent inflammation, as well as hypoxia, can delay wound healing and increase the risk of pathological scar formation, ischemia, and necrosis.^103,104^ HBOT improves tissue oxygenation and fibroblast function, promotes neovascularization, and can markedly decrease ischemia reperfusion injury in skin flaps.^73,78^ The authors recognize that HBOT is neither accessible nor necessary for all patients; however, they suggest a consideration of HBOT for high-risk patients presenting with vascular compromise and/or excessive fibrosis to improve flap viability and improved outcomes.

### Lymphatic Massage

Lymphatic massage has been proven to reduce pain, swelling, and fibrosis in patients undergoing liposuction or lipoabdominoplasty.^105^ Additional data suggest that lymphatic massage may also have beneficial effects on the arteriovenous system, increasing peripheral arterial blood flow through superficial or skin circulation.^105^ Lymphatic massage has thus been incorporated into the postoperative recovery for various cosmetic procedures and is recommended by the authors for patients with a history of MITs, prolonged edema, and vascular compromise.^105^

### Systemic Steroids

Initiation of postoperative systemic steroids may be particularly beneficial in patients with a history of MITs, especially those who have previously exhibited intense or prolonged inflammatory responses. Early administration of systemic steroids might help to modulate the duration and severity of inflammation, potentially leading to improved healing, a reduced risk of complications, and more favorable surgical outcomes. Careful consideration of the patient's history and inflammatory response patterns should guide the decision to use systemic steroids, including the dosage, in the postoperative period and be balanced with the potential risks. Additionally, 1 author suggests that alternative immune modulators may be considered, depending on the patient's clinical profile and preferences.

### Summary

The increasing prevalence of MITs in aesthetic medicine necessitates a comprehensive understanding of their potential impact on subsequent facial surgery. The authors of this paper provide valuable insights and evidence-based recommendations to guide both patients and practitioners in optimizing outcomes when aesthetic surgery follows MITs; however, individual approaches should be utilized to mitigate risk associated with previous MITs until further research is available.

The experience and literature findings emphasize that although MITs do not inherently preclude future aesthetic facial surgery, they introduce potential complications that can influence surgical planning and outcomes. The most critical issues arise from tissue plane distortion and vascular compromise, particularly in patients treated with deeply delivered EBDs or improperly administered injectables. These complications can challenge the surgeon's ability to perform precise dissections, maintain flap viability, and achieve the desired aesthetic results.

To mitigate these risks, the expert panel has recommended a series of perioperative strategies. These include thorough preoperative assessments, careful timing of surgery relative to the last MIT, and specific intraoperative techniques tailored to address altered tissue planes and compromised vascularity. Additionally, postoperative care should be individualized, with focused management of inflammation, fibrosis, and potential vascular issues.

Limitations to these recommendations include the constantly changing technology and adjunctive treatments. Recommendations made in this study are reflective of the available technology and treatments at the time of publication. In addition, the opinions are of the participating expert panelist and clinical judgment should be followed for each individual case by the operating surgeon.

## CONCLUSIONS

In conclusion, as MITs continue to rise in popularity, the interplay between these treatments and subsequent facial surgery must be carefully considered, and further research is needed to deepen understanding and optimize management strategies. By adhering to the guidelines and strategies outlined herein, surgeons and aesthetic providers can better navigate the complexities introduced by MITs, ultimately enhancing patient safety and surgical outcomes.

## Supplementary Material

ojaf087_Supplementary_Data
