# Supplementary material for: Optimizing Aesthetic Facial Surgery Outcomes Following Minimally Invasive Treatments: Guidelines for Perioperative Management
Source: Aesthet Surg J Open Forum. 2025 Jul 4;7:ojaf087. doi: 10.1093/asjof/ojaf087 (PMC12368962; doi:10.1093/asjof/ojaf087)
Supplement: ojaf087_Supplementary_Data [file ojaf087_supplementary_data.zip › Supplement Figure.docx]

**Supplement Figure:** Algorithm for Perioperative Management of Patients with a History of MITsThis algorithm outlines a stepwise approach to preoperative, intraoperative, and postoperative planning for aesthetic facial surgery in patients with a history of MITs. The algorithm includes specific recommendations based on treatment modality (e.g., injectables, threads, energy-based devices) to address challenges such as tissue plane distortion and vascular compromise.5-FU, fluorouracil; EBD, energy-based device; HA, hyaluronic acid; MIT, minimally invasive treatment.
