# Supplementary figures and images for: Optimizing Aesthetic Facial Surgery Outcomes Following Minimally Invasive Treatments: Guidelines for Perioperative Management
Source: Aesthet Surg J Open Forum. 2025 Jul 4;7:ojaf087. doi: 10.1093/asjof/ojaf087 (PMC12368962; doi:10.1093/asjof/ojaf087)

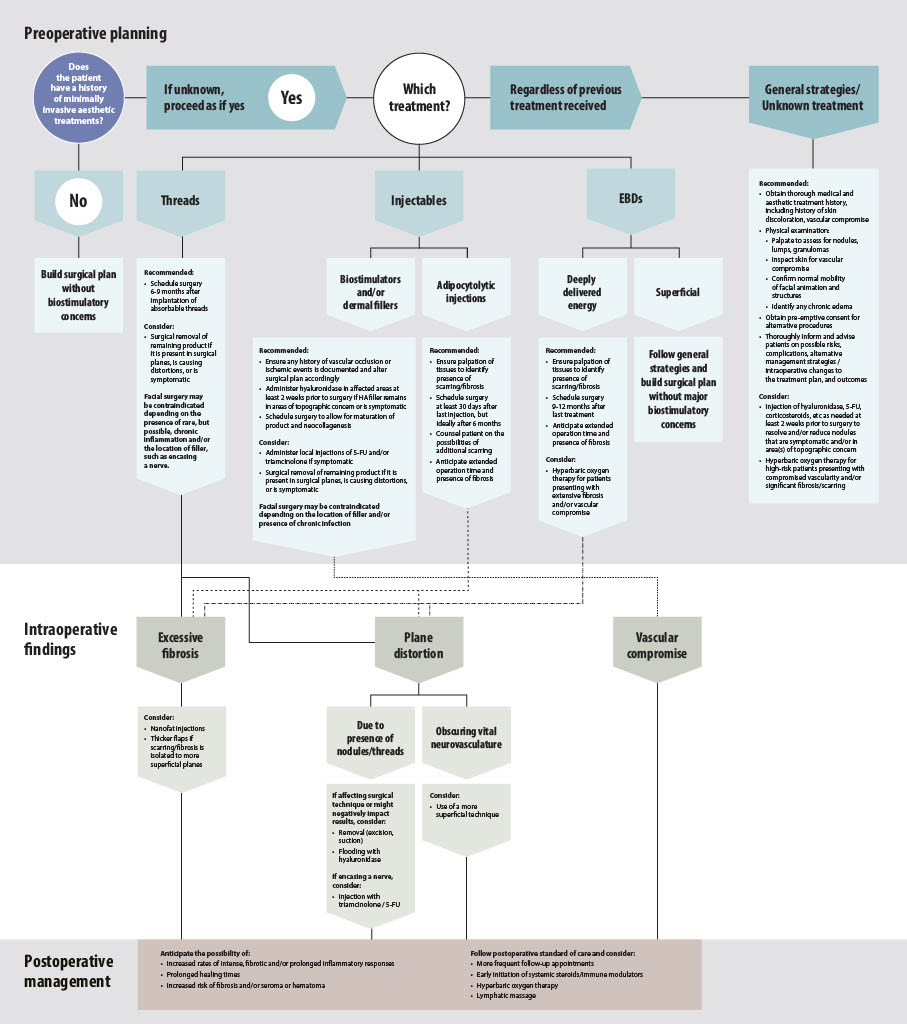

Supplement: ojaf087_Supplementary_Data [file ojaf087_supplementary_data.zip › Supplement 2 Algorithm.jpg]
